# PW02-003 - Efficacy of anakinra in etanercept-resistant TRAPS

**DOI:** 10.1186/1546-0096-11-S1-A143

**Published:** 2013-11-08

**Authors:** AK Ombrello, PM Hoffmann, A Jones, KS Barron, DL Kastner

**Affiliations:** 1 NHGRI, National Institutes of Health, Bethesda, USA; 2 NIAID, National Institutes of Health, Bethesda, USA

## Introduction

Tumor necrosis factor receptor-associated periodic syndrome (TRAPS) is an autoinflammatory disease inherited in an autosomal dominant fashion. TRAPS develops secondary to mutations in *TNFRSF1A.* Associated symptoms include periodic attacks of peritonitis, constipation, arthritis in large joints, arthralgia, migratory rash with underlying myalgia, periorbital edema, conjunctivitis, splenomegaly, and increased risk for AA amyloidosis. Typically, attacks last from days to weeks. The common treatment modalities are corticosteroids, the p75 TNFR:Fc fusion protein, etanercept, and IL-1 antagonists. Recent studies suggest that multiple cytokines are involved in the pathogenesis of TRAPS. To date, there are limited data comparing the efficacy of etanercept and IL-1 inhibitors in TRAPS.

## Objectives

To explore the efficacy of anakinra in nine TRAPS patients who had modest response while on etanercept.

## Methods

CRP and ESR were measured serially in nine patients with TRAPS (eight adults and one child) who had been initially treated with etanercept and were subsequently switched to anakinra. Patient records were evaluated for clinical and laboratory associations. Patients with the R92Q and P46L variants were excluded from our analysis.

## Results

Eight adult patients and one child with TRAPS were switched from etanercept to anakinra treatment due to poor symptom control and persistent elevation in inflammatory markers. Among all nine patients, the range of ESR before starting anakinra (no patients were actively flaring at the time labs were drawn) was 37-91 mm/hr and after was 5-18 mm/hr. The range of CRP before starting anakinra was 18.10-186 mg/L and after was <0.5-8.85 mg/L. The etanercept doses ranged from 50 mg weekly to 75 mg weekly. The anakinra doses ranged from 100 mg daily to 300 mg daily. One patient with AA amyloidosis had normalization of proteinuria and stabilization of creatinine within 16 months of starting anakinra. Patients reported fewer flares, shorter duration of flares, and decreased necessity for additional medications during flares (corticosteroids and narcotics).

## Conclusion

Our findings indicate that in some patients, anakinra is superior to etanercept for the treatment of TRAPS. Of the nine patients, all of them experienced clinically significant decreases in inflammatory markers including CRP and ESR as well as clinical improvement in symptoms related to TRAPS upon the initiation of anakinra.

## Disclosure of interest

None declared.

